# L'appendagite épiploïque primitive: une cause rare de douleur abdominal

**DOI:** 10.11604/pamj.2015.20.170.5638

**Published:** 2015-02-24

**Authors:** Issam Yazough, Hecham El bouhadouti, Khalid Mazaz

**Affiliations:** 1 Faculté de Médecine et de Pharmacie de Fès, Université Sidi Mohammed Ben Abdellah, Département de Chirurgie, CHU Hassan II, Fès, Maroc

**Keywords:** Appendagite, épiploïque, primitive, appendagitis, omental, primitive

## Abstract

Les appendagites sont une cause rare de douleur abdominale chez l'adulte, elles simulent souvent le tableau d'une appendicite aiguë ou une sigmoïdite, le diagnostic est souvent poser par une TDM ou lors d'un acte chirurgicale, le traitement est essentiellement médicale.

## Introduction

L'appendagite est une des causes de douleur abdominale chez l'adulte; c'est un diagnostic différentiel de l'appendicite qui doit être évoqué, car son traitement est conservateur.

## Patient et observation

**Figure 1:**
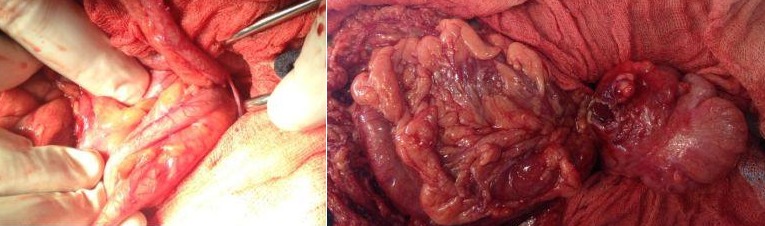
Image en per-opératoire d'un appendice

Patient âgé de 33 ans, opéré il ya 6 ans pour un remplacement valvulaire fut admis aux urgences dans un tableau de douleur de la fosse iliaque droite, avec l'examen une défense de la fosse iliaque droite subfébrile a 37,8 le bilan biologique a révélé une hyperleucocytose a 14 000, une échographie abdominale a été en faveur d'une infiltration de la graisse au niveau de la fosse iliaque droite sans individualisation d'appendice et une lame d’épanchement, la décision était d'admettre le patient au bloc opératoire, dont l'exploration a mis en évidence un appendice normale, avec une frange caecale nécrosée colmatée par le grand épiploon (Figure 1).

## Discussion

L'appendagite est une étiologie de douleur abdominale rare chez l'adulte [1], l'appendagite épiploïque primitive, complication la plus fréquente, correspond à l'inflammation d'un appendice épiploïque par torsion ou spontanément par thrombose de la veine de drainage de l'appendice épiploïque. L'appendagite épiploïque secondaire se développe suite à un processus inflammatoire entreprenant une structure adjacente comme un diverticule ou l'appendice. Cliniquement, cette pathologie se manifeste par une douleur abdominale localisée qui peut mimer une appendicite iléo-caecale ou une diverticulite. Aussi, par le passé, il n’était pas rare que le diagnostic apparaisse lors de la laparotomie. Cependant l'avènement de l'utilisation de la tomodensitométrie dans l’établissement du diagnostic des douleurs abdominales permet l'identification de l'appendagite épiploïque par des signes radiologiques pathognomoniques, minimisant le risque d'une chirurgie ou d'une hospitalisation inutiles. Aussi, selon des séries de cas récents, l'appendagite épiploïque primitive serait le diagnostic correct dans 2 à 7% des cas présumés de diverticulite et dans 1% des cas présumés d'appendicite [2, 3]. L'aspect échographique le plus souvent observé chez l'adulte est celui d'un nodule, non compressible et antérieur par rapport au colon [4]. L’étude en doppler couleur révèle une absence ou une baisse de flux au sein de la masse et une hypervascularisation périphérique. L'exploration TDM, permettant de faire le diagnostic de certitude, se traduit par l'existence d'une masse ovalaire adjacente au colon de densité plus élevée que la graisse péritonéale normale (-53 U.H) [5]. Le signe constamment retrouvé est celui du «ring sign» se traduisant par une hyperdensité en périphérie de la lésion correspondant à l'inflammation de la séreuse [6]. Moins fréquemment, le scanner peut révéler [7]: une hyperdensité punctiforme centrale spontanée (54%) en rapport avec la thrombose de la veinule centrale; un épaississement du péritoine pariétal et viscéral; un épaississement des anses adjacentes. La durée d’évolution clinique de la maladie est en moyenne de 4 jours [8]. Le traitement conservateur antalgique de première intention après diagnostic précoce à l'imagerie est le traitement de référence [2]. La régression des signes radiologiques varie entre 2 semaines et 6 mois et il peut persister des stigmates fibreuses ou des calcifications [8, 5, 7].

## Conclusion

L'apparition brutale d'une douleur abdominale focale quasiment monosymptomatique dans un des quadrants inférieurs de l'abdomen doit faire évoquer le diagnostic d'appendagite épiploïque. La tomodensitométrie abdomino-pelvienne apportera le diagnostic permettant d’éviter des laparotomies et des hospitalisations excessives.
